# Electrochemical and Optical Sensors for the Detection of Chemical Carcinogens Causing Leukemia

**DOI:** 10.3390/s23073369

**Published:** 2023-03-23

**Authors:** Adrian Kowalczyk, Julia Zarychta, Monika Lejman, Joanna Zawitkowska

**Affiliations:** 1 Student Scientific Society of Department of Pediatric Hematology, Oncology and Transplantology, Medical University, 20-093 Lublin, Poland; 2 Independent Laboratory of Genetic Diagnostics, Medical University of Lublin, 20-093 Lublin, Poland; 3 Department of Pediatric Hematology, Oncology and Transplantology, Medical University, 20-093 Lublin, Poland

**Keywords:** leukemia, chemical carcinogens, electrochemical sensors

## Abstract

The incidence and mortality due to neoplastic diseases have shown an increasing tendency over the years. Based on GLOBOCAN 2020 published by the International Agency for Research on Cancer (IARC), leukemias are the thirteenth most commonly diagnosed cancer in the world, with 78.6% of leukemia cases diagnosed in countries with a very high or high Human Development Index (HDI). Carcinogenesis is a complex process initiated by a mutation in DNA that may be caused by chemical carcinogens present in polluted environments and human diet. The IARC has identified 122 human carcinogens, e.g., benzene, formaldehyde, pentachlorophenol, and 93 probable human carcinogens, e.g., styrene, diazinone. The aim of the following review is to present the chemical carcinogens involved or likely to be involved in the pathogenesis of leukemia and to summarize the latest reports on the possibility of detecting these compounds in the environment or food with the use of electrochemical sensors.

## 1. Introduction

The incidence and mortality due to neoplastic diseases have shown an increasing trend over the years. Leukemias are a heterogeneous group of malignant neoplasms of the hematopoietic system, in which the clonal expansion of neoplastic cells occurs in the bone marrow and in the blood [1,2]. The disease is caused by disorders in the process of the production and differentiation of morphotic blood elements in the myeloid or lymphoid lines [1]. The classic division of leukemias includes: acute myeloid leukemia (AML), acute lymphoblastic leukemia (ALL), chronic myeloid leukemia (CML), and chronic lymphocytic leukemia (CLL) [2]. ALL is the most common type of leukemia in children [3]. The incidence of ALL decreases in direct proportion to age [3]. The adult population has a higher incidence of chronic types of leukemia, and men are reported to be more likely to suffer from it due to the increased occupational exposure to carcinogens [4].

Based on GLOBOCAN 2020 published by the International Agency for Research on Cancer (IARC), leukemias are the thirteenth most commonly diagnosed cancer in the world. In 2020, 474,519 cases of leukemia were diagnosed. Leukemia was the tenth leading cause of death from cancer, accounting for 311,594 deaths. In 2020, 78.6% of leukemia cases were diagnosed in countries with a very high or high Human Development Index (HDI) [5]. In 2020, the incidence of leukemia was the highest in North America, Australia, and New Zealand, followed by Europe and Western Asia. Available studies indicate a higher incidence of leukemia in industrialized, highly developed countries, which may be caused by both better access to the health care system and greater environmental pollution [1,3,6,7]. The exception is ALL, which is most common in South and Central America [1].

Numerous compounds present in the environment are considered carcinogenic: the IARC has identified 122 human carcinogens (group 1) and 93 probable human carcinogens (group 2A) [8]. Exposure to toxic compounds promotes the development of mutations. On the other hand, mutated cells expressing oncogenes are more sensitive to the action of mutagenic compounds, which causes the occurrence of subsequent mutations and their accumulation, ultimately leading to a neoplastic transformation [9,10]. Among groups 1 and 2A of carcinogens identified by the IARC, we focused in our work on chemical carcinogens that affect the hematopoietic system and thus may have an impact on the development of leukemia. Chemical carcinogens described by the IARC as having the potential to cause leukemia based on sufficient evidence are listed in Table 1.

Most chemical carcinogens share a common mechanism of action. Entering the electrophilic form, they can bind to nucleophilic sites, which include, among others, purine and pyrimidine rings of nucleic acids. In order to enter the electrophilic form, some chemical carcinogens must undergo a metabolic transformation catalyzed by cytochrome P450 or CYP enzymes. The effect of the interaction of chemical carcinogens with DNA is the appearance of mutations that can initiate or cause the progression of carcinogenesis [10].

The identification of toxic compounds in the environment and food products as well as monitoring their concentration in the air in real time, using a miniaturized and easy-to-use sensor, can contribute to the increase in social awareness related to the concentration of specific pollutants to which people are exposed in everyday life. This in turn could enable people to take effective measures to prevent exposure to toxic compounds in the workplace and/or at home, as well as increasing the safety of consumed food.

The following review aims to discuss the chemical carcinogens involved or likely to be involved in the pathogenesis of leukemia and to synthesize the latest reports on the possibility of detecting these compounds in the environment or food with the use of electrochemical or optical sensors or biosensors. In electrochemical sensors the target compound interacts with an electrolyte, which leads to electrochemical reactions producing a current which can be measured [11]. These sensors have gained great popularity, because they are fast, selective, sensitive, and easy-to-use analytical tools. Their ease of miniaturization and integration into automatic systems, without compromising their analytical characteristics, have been met with wide interest [12]. The main advantage of optical sensors over electrochemical sensors is that the results could be easily identified by the naked eye [13].

## 2. Electrochemical Sensors for the Detection of Chemical Carcinogens Causing Leukemia

### 2.1. Benzene

#### 2.1.1. Benzene Overview

Benzene (C_6_H_6_, CAS 71-43-2) is a colorless, volatile liquid at room temperature, and highly flammable aromatic hydrocarbon [14]. It is released during natural processes such as forest fires, volcanic eruptions, but also because of human activity [15]. The main sources of benzene resulting from human activity are: car exhaust fumes, car refueling, and industrial emissions [16]. In 2016, world production of benzene was 44.9 million tonnes, of which more than 55% was in the Asia-Pacific region [17]. Benzene concentrations in the environment range from 0.2 µg/m^3^ in rural areas to 349 µg/m^3^ in industrial centers with heavy car traffic [16]. 

Benzene enters the body mainly through inhalation, and the average percentage of absorption in this way oscillates from about 50 to 80%. After absorption, it quickly disperses in all tissues [15]. Benzene is metabolized in the liver, where it is biotransformed in the presence of cytochrome P450, mainly CYP 2E1. The epoxidation process produces benzene oxide and oxepine. Benzene oxide is further oxidized to phenol and then hydroquinone, or is converted in the presence of epoxide hydrolase to benzene dihydrodiol and further to catechol. These products are transported by blood to the bone marrow, where under high concentrations of myeloperoxidase, hydroquinone is converted into 1,4-benzoquinone (1,4-BQ), and catechol into 1,2-benzoquinone [18].

Benzene concentration limits are set differently in various countries. Namely, the Occupational Exposure Limit set by the European Chemical Agency is 1 ppm (3.25 mg/m^3^) and it is followed by most European countries, although some of them set lower values, e.g., Poland and Denmark—1.6 mg/m^3^. Meanwhile, in the USA the 8 h permissible exposure limit and short-term limit set by the Occupational Safety and Health Administration are 1 ppm (3.19 mg/m^3^) and 5 ppm (15.95 mg/m^3^), respectively. The benzene air concentration limit is 5 µg/m^3^ in Europe, but the World Health Organization (WHO) states that there is no safe level of exposure to benzene [19].

One of the latest editions of the IARC monograph from 2018 confirmed that benzene is carcinogenic to humans and distinguished various types of leukemia associated with benzene exposure. It was argued that there is sufficient evidence for a link between benzene and AML in adults. In addition, positive correlations were observed for CLL, CML, non-Hodgkin lymphoma, multiple myeloma, lung cancer, and AML in children. The mechanism of action of benzene was also analyzed, and it was found out that there is strong evidence that this compound is activated in the body by electrophilic metabolites, causing, i.a., oxidative stress and DNA damage, chromosomal changes, and immunosuppression [19]. For possible leukemia-inducing mechanisms of action of benzene, see Table 2.

#### 2.1.2. Sensors for Benzene Detection

According to the European Air Quality Directive, the reference method for measuring benzene, toluene, ethylbenzene, and xylene (BTEX) gases is sampling followed by gas chromatography [20]. This method is perceived as accurate, but often expensive, bulky, and time-consuming. In addition, it requires complex sample preparation [21,22,23]. Therefore, for real-time detection, low-cost and portable sensors are used. Various types of sensors for benzene monitoring are currently available on the market, e.g., metal oxide sensors, amperometric or potentiometric cells, photo-ionization detectors, portable and micro gas chromatography. However, they have some disadvantages. Namely, amperometric electrochemical sensors are not able to achieve low levels of benzene detection, photo-ionization detectors have poor selectivity, and portable gas chromatographs are very expensive [20]. Therefore, continuous research is being carried out on low-cost sensors with high selectivity and sensitivity.

**Figure 1:**
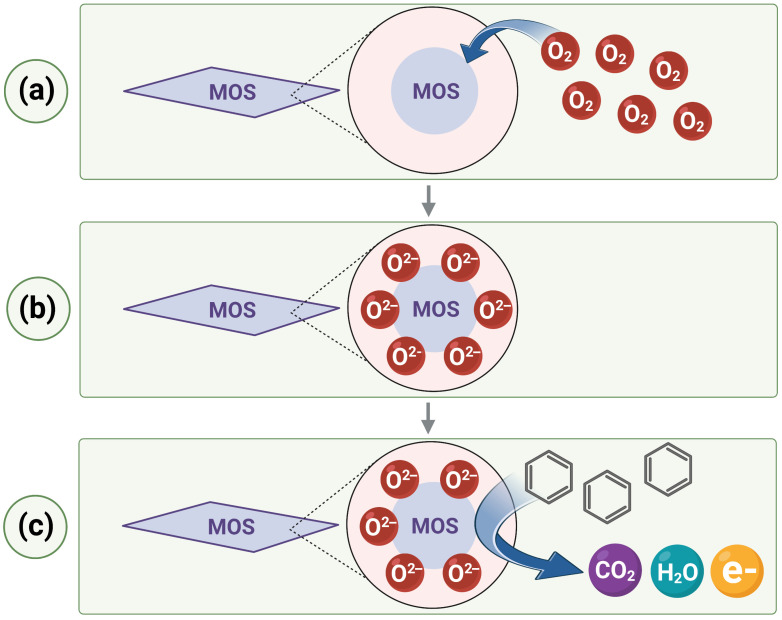
The mechanism of the operation of sensors based on MOS: (**a**) In air environment, oxygen is adsorbed onto the surface of MOS; (**b**) oxygen adsorption creates a surface layer of negatively charged oxygen ions; (**c**) when benzene is present, it is oxidized by negatively charged oxygen ions with the release of electrons, which results in the change in resistance and makes it possible to detect benzene. Image created with biorender.com (accessed on 15 February 2023).

A popular strategy in the design of sensors for the detection of benzene is the use of metal oxide semiconductors (MOSs). The MOS detection mechanism consists in the absorption of oxygen on the surface of the oxides followed by the reaction with the tested gas. This reaction triggers the release of electrons, changing the resistance of the sensor. These changes make it possible to detect the sought gas (see Figure 1) [24]. 

One of the problems related to detecting benzene concerns the similarity between benzene and other volatile organic compounds (VOCs), but it could be solved by the application of catalytic filters. In one study, a combination of a Pd/SnO_2_ sensor with a catalytic filter of WO_3_ nanoparticles, designed to prevent interference from other VOCs, was made. Each VOC reacts with WO_3_ at a different temperature, therefore, at a temperature of about 240 °C, the catalyst completely removes m-xylene and toluene, thus enabling the selective detection of benzene [21]. Another example of a reported catalytic filter is Rh-TiO_2_. The sensory function in this case was performed by a SnO_2_ layer placed on an Au electrode. Then, the behavior of this sensor after coating the SnO_2_ layer with 0.5, 1, and 2 wt% Rh-TiO_2_ was investigated. It turned out that the sensor coated with 2 wt% Rh-TiO_2_ enabled the highly selective detection of benzene at 325 °C with negligible interference from other air pollutants. The authors explain this by the oxidative filtering of other interfering gases with higher reactivity than benzene, which is very stable due to its structure [25]. In another study, a Co_3_O_4_ overlayer was used as the catalytic filter and underneath it there was Pd-SnO_3_ sensing film, the thickness of which was ~25 μm. This means that the sensor response depends on the transfer of the analyzed gas to the bottom of the sensor layer near the electrodes. This structure makes all gases more reactive than benzene oxidize in the upper layers to non-reactive or less reactive gases, i.e., CO_2_ and H_2_O. On the other hand, benzene, on its way to the lower layers of the sensor, is transformed into smaller and more active molecules, which enables its selective detection [22]. Another way to improve the selectivity of benzene over other VOCs can be molecular imprinting (MIP). In one study the researchers used this technique to modify a Ag-LaFeO_3_ sensor with p-type boron-doped graphene quantum dots (BGQDs) by means of benzene imprinting. The sensor constructed in this way (B/APPH) was tested and compared to non-imprinted Ag-LaFeO_3_ (NI-AL) and benzene-imprinted Ag-LaFeO_3_ (BI-AL). BI-AL showed promising results in detecting benzene, and after connecting it to the BGQDs, it was possible to reduce the operating temperature to 65 °C [24]. 

In one paper, the researchers point out that the efficiency of gas detection can be improved by increasing the detection area, e.g., by preparing nanostructures with a high surface to volume ratio. For this purpose, the authors decided to use branched nanowires (NWs), which were prepared with TiO_2_ branches grown on Si stem NWs. The NW stem–branch sensor constructed in this way has many resistance elements involved at the points of interaction with the gas: the surface depletion layers along the stem and branch NWs, the networked homojunctions, and the stem–branch heterojunctions, and the latter in particular increased the detection of benzene [26].

In another work, the authors point out that in metal oxide-based sensors, chemical reactions are thermally activated. High temperature, provided by external heaters, increases the sensitivity of the sensor to a specific gas. However, it results in high energy consumption and, therefore, in this form the possibility of using these sensors on a large scale seems unlikely. One way to overcome this problem is to use a self-heating strategy. An example of a sensor using this phenomenon, based on Pd-functionalized SnO_2_–ZnO core–shell NWs (C-S NWs), was presented, both SnO_2_ and ZnO being popular choices for gas sensors. Combining them in the form of core–shell NWs synergistically improves their ability to detect gases. On the other hand, Pd, due to its catalytic activity towards benzene, can additionally strengthen the properties of the sensor. The self-heating effect is achieved by applying a voltage to record the resistance of the sensor. The heat in this case is generated as a result of the loss of kinetic energy of electrons when collisions occur between the transferred electrons and the atoms of the crystal lattice. Increasing the applied voltage leads to an increased heat production, and this can be used to reduce the energy consumption of the gas sensor [27]. Another method to overcome this problem is to modify the MOS with substances working at room temperature. The possibility of constructing a benzene sensor using a Pd-decorated TiO_2_/MoS_2_ ternary nanocomposite as a sensing material has been presented in one paper. TiO_2_ is widely used as a semiconductor in sensors for detecting various gases, but it has some disadvantages such as low selectivity, high working temperature, and high power consumption. The authors emphasize that the modification of TiO_2_ with noble metal and graphene-based nanomaterials can enhance the ability of TiO_2_ to detect gases. In recent years, transitional metal dichalcogenides, including molybdenum disulfide (MoS_2_), with a structure similar to graphene and a possibility to work at room temperature, have attracted attention as semiconductors. This work presents a sensor based on TiO_2_ decorated with Pd, modified with MoS_2_. The operation mechanism of this sensor consists in the absorption of oxygen molecules on the surface of TiO_2_, which are converted to negatively charged ions. When the sensor is exposed to benzene, negatively charged oxygen ions react with benzene molecules. In this reaction, electrons are released, which reduces the resistance of the electrode and makes the detection of benzene possible. The sensor constructed in this way showed high sensitivity, fast response/recovery behavior, and low detection limit [28]. In one study, the ability to detect benzene at room temperature by nanostructured thin films of ZnO-CdO mixed oxide was investigated. Three different ratios of ZnCl_2_ and CdCl_2_ were considered in this work: 0.08 M:0.02 M, 0.06 M:0.04 M, and 0.04 M:0.06 M. The results of these three different layers were compared with pure zinc oxide. In the air environment, oxygen molecules adsorb onto the surface of mixed oxides. Oxygen molecules, by consuming electrons, make the space charge width widen and in this way they increase the surface resistance. When benzene enters the sensor chamber, it reacts with the absorbed oxygen molecules, undergoing reduction and releasing electrons in the process. This leads to a decrease in the surface resistance. All mixed oxide thin films were selective for benzene vapors, but the layer deposited in the ratio 0.04:0.06 M was characterized by a particularly high selectivity [23].

Although MOS-based sensors are very popular, they have some disadvantages such as high power consumption, short lifetime, and a limited working temperature range. Therefore, research is underway to develop other benzene detection strategies. In one work, a sensor based on covalent organic frameworks (COFs) was constructed [29]. COFs are a class of porous materials, which are created through the integration of organic precursors into complex structures with periodic skeletons and ordered pores [30]. This sensor was created by the reaction between benzene-1,3,5-tricarboxaldehyde (BTA) and 2,4,6-tris(4-aminophenyl)-1,3,5-triazine (TAPT). The resulting sensor—BTA-TAPT COF—had advantages such as good crystallinity, large specific surface area, and high thermal stability. The sensor constructed in this way had a detection limit of 340 ppb as well as lower power consumption and better selectivity compared to conventional MOS-based sensors [29].

In one study an organic–inorganic layered perovskite compound, (C_4_H_9_NH_3_)_2_PbI_2_Br_2_, was developed. It achieved very good results in the detection of benzene down to the ppt level. The interaction of benzene with this sensor results in the presence of characteristic peaks measured with diffuse reflectance Fourier transform infrared spectroscopy, which proves the adsorption of benzene molecules on the surface of (C_4_H_9_NH_3_)_2_PbI_2_Br_2_. The authors point out that organic–inorganic hybrid perovskites combine the excellent performance of organic materials and inorganic semiconductors, and are a low-cost option for sensor designing [31].

One work reported the construction of an ionogel (IO)-based sensor for the detection of formaldehyde and benzene. The sensory layer was created by using a 1-ethyl-3-methylimidazolium ethyl sulfate ([C_2_mim][EtSO_4_]) ionic liquid (IL) encapsulated into a poly(*N*-isopropylacrylamide) (pNIPAAM) matrix. The sensitivity of this device for detecting benzene and formaldehyde was tested in the presence of two carrier gases (N_2_ and synthetic air), then changes in resistance were compared with the “pure” ionic liquid. More sensitivity was demonstrated in the detection of benzene using the ionogel-based technique than when using the “pure” ionic liquid. It also turned out that IO showed a greater affinity for benzene, which was explained by interactions between imidazole cations and benzene molecules. This resulted in greater sensitivity and a lower limit of detection [32].

One paper demonstrated the possibility of using odorant-binding proteins (OBPs) to detect benzene in outdoor air in real time. For this purpose, a biosensor was created in which porcine OBPs isolated from pigs were placed on the surface of gold interdigitated electrodes. These particles, immobilized on the working electrode (WE), act as a molecular recognition element. When they interact with benzene, a change in the capacitance value is measured. The sensor constructed in this way was able to detect benzene in the air at a concentration of 64 pM (5 µg/m^3^) [33]. For the linear range and the detection limits of SnO_2_/Rh-TiO_2_, Pd-decorated TiO_2_/MoS_2_, ZnO-CdO, (C_4_H_9_NH_3_)_2_PbI_2_Br_2_, and IL/pNIPAAM, see Table 3.

### 2.2. Formaldehyde

#### 2.2.1. Formaldehyde Overview

Formaldehyde (CH_2_O, CAS: 50-00-0) is an organic chemical compound, well soluble in water. At room temperature, it is a colorless gas [34,35,36,37]. The WHO established in its guidelines that the concentration of formaldehyde in the air should be kept below 0.1 mg/m^3^/0.08 ppm [38].

Formaldehyde is widely distributed in the environment into which it is released during natural processes and anthropogenic human activity [34,39,40]. The concentration of formaldehyde in closed rooms is higher than in open spaces because it is released from building materials containing formaldehyde-based resins, for example, from wooden plywood, which can cause sick building syndrome [34,36,37,41,42,43]. Another source of formaldehyde is cooking, in particular frying [34,36,44]. Due to its bactericidal properties, formaldehyde is used in the production of formalin [45,46,47]. Formaldehyde can be released from tattoo inks, eyelash glues, and preservatives used in the production of personal hygiene lotions, nail polishes, makeup removers, lotions, and creams [42,48,49,50]. Formaldehyde has also been detected in cigarette smoke and e-liquid from electronic cigarettes [34,51]. The compound is a by-product of the poly(ethylene terephthalate) synthesis process, used for the production of beverages, e.g., beer, and food packaging, into which it can migrate [44,52]. It is also a by-product of water treatment processes [53,54]. Formaldehyde is used illegally as a preservative to extend shelf life in products (seafood, fish, milk, dairy products, fruits, and vegetables) [55,56,57]. 

Formaldehyde enters the body mainly during breathing and it is absorbed in the upper and lower respiratory tract, where it is rapidly metabolized, thanks to which its concentration in the blood is relatively constant [34,39,45,58]. A small amount of formaldehyde penetrates the body through the skin and is ingested with food and drink [41,59]. Formaldehyde is an electrophile and may have a mutagenic effect on cells due to its ability to form covalent bonds with nucleophilic sites found in proteins and nucleic acids [41,60]. Formaldehyde metabolism causes oxidative stress by increasing the level of reactive oxygen species (ROS) and reducing the expression of the *PRDX2* gene, which encodes an antioxidant enzyme, contributing to cell damage [41,60]. Formaldehyde reduces the expression of *GM-CSFRα* and *EPOR* genes, interfering with the activity of hematopoietic growth factors [61]. One study showed that exposure to formaldehyde increases the risk of chromosomal aberrations occurring in the course of AML (chromosome 7 monosomy and chromosome 8 trisomy); however, the methodology of the study is now questioned and the need to repeat it is indicated [62,63,64,65,66]. The IARC classified formaldehyde as a human carcinogen (group 1), capable of inducing nasopharyngeal cancer (2004) and leukemia (2012), especially AML [8,39,45,47,58]. Despite the toxic effect of formaldehyde on cells, the mechanism by which it can induce leukemia is unclear [39,58]. Potential mechanisms have been proposed in which formaldehyde may contribute to the development of leukemia: direct damage to bone marrow stem cells, damage to hematopoietic stem cells circulating in the blood or present in the nasal passages, which are then transported with the blood to the bone marrow and initiate the development of cancer [39,62,66]. For possible leukemia-inducing mechanisms of action of formaldehyde, see Table 2.

#### 2.2.2. Sensors for Formaldehyde Detection

Traditional formaldehyde detection techniques include: gas chromatography, high-performance liquid chromatography, or colorimetry-based derivatization [67]. Electrochemical and optical sensors based on colorimetric technology are available on the market for real-time formaldehyde detection. The colorimetric technique is characterized by greater sensitivity and selectivity, but the production of colorimetric sensors is usually more expensive than in the case of electrochemical sensors. In addition, colorimetric devices require a long acquisition time (at least 30 min). On the other hand, the disadvantage of electrochemical sensors is that they interfere with other compounds, in particular alcohol [68]. Therefore, research is being conducted on the further development of these technologies and the use of other methods of the detection of formaldehyde.

**Figure 2:**
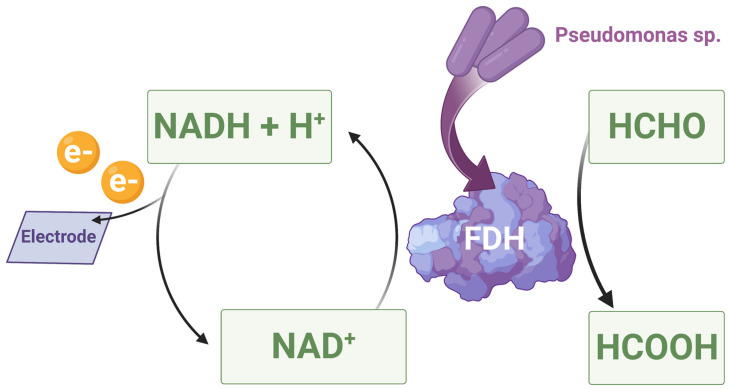
The mechanism of the operation of biosensors based on FDH derived from Gram-negative bacteria *Pseudomonas* sp.: FDH present in the biosensor oxidizes formaldehyde, resulting in the reduction of nicotinamide adenine dinucleotide (NAD+) to hydrogenated nicotinamide adenine dinucleotide (NADH) with an accompanying transfer of electrons, which make it possible to detect formaldehyde. Image created with biorender.com (accessed on 10 March 2023).

One of the other possibilities for formaldehyde detection is the use of electrochemical biosensors based on oxidoreductases. A direct electron transfer biosensor was developed using formaldehyde dehydrogenase (FDH) derived from Gram-negative bacteria *Pseudomonas* sp. to specifically oxidize formaldehyde to formic acid. The oxidoreductase was placed on a gold electrode previously covered with a solution of gold nanoparticles (AuNPs). Formaldehyde is oxidized by oxidoreductase, resulting in the reduction of nicotinamide adenine dinucleotide (NAD^+^) to hydrogenated nicotinamide adenine dinucleotide (NADH) with an accompanying transfer of electrons (see Figure 2). There is an increase in the anodic current and a change in the cyclic voltammetry (CV) curve, making it possible to detect the compound [69]. 

Another self-powered electrochemical biosensor based on FDH detects gaseous formaldehyde by using an electrochromatic display that changes color when a current is applied. On an indium tin oxide (ITO) substrate, a Prussian blue (BP) film was placed and acted as a cathode, whereas formaldehyde dehydrogenase/poly(methylene green)/buckypaper (FDH/PMG/BP) placed on ITO acted as a bioanode. FDH present in the bioanode oxidizes formaldehyde, releasing an electron, which is accepted by the cathode, and the blue color becomes colorless. Depending on the concentration of gaseous formaldehyde, the color front moves away from the bioanode towards the cathode, reflecting the amount of electrons released during the reaction catalyzed by FDH. An optical analysis of a sensor photo by appropriate software on a smartphone may also enable the quantitative determination of formaldehyde concentration in the air in the future [70]. Another biosensor using FDH can detect formaldehyde in fruit juices. Using the electrophoretic deposition technique (EPD), on a glass electrode coated with ITO, multi-walled carbon nanotubes (CNTs) and the CNT-Fe_3_O_4_ nanocomposite were deposited, yielding two electrodes: CNT/ITO and CNT-Fe_3_O_4_/ITO. Cyclic voltammetric tests of bioelectrodes were applied in order to illustrate the oxidation and reduction peaks obtained for different concentrations of formaldehyde in the tested electrolyte, with the relative change in current being more pronounced in the case of the BSA/FDH/CNT-Fe_3_O_4_/ITO bioelectrode [55]. FDH is not the only enzyme used in the production of biosensors that detect formaldehyde. An optical biosensor using the enzyme alcohol oxidase (AOx) was also designed. The biosensor consists of a glass slide on which a pH-sensitive MB28 copolymer membrane is placed, and a sol–gel membrane in which AOx is trapped. The enzyme catalyzes the formaldehyde oxidation reaction, in which hydrogen peroxide and formic acid are formed, dissociating the formate anion and hydrogen cation. The hydrogen cation then goes to the proton-selective layer of the MB28 membrane, where it forms an ion-chromoionophore (HNBCM^+^) with the Nile Blue chromoionophore (NBCM) present in the MB28 layer. The formation of HNBCM^+^ leads to the change in the color of the sensor from light blue to dark blue. Measuring the absorption of light by membranes using a UV–Vis spectrophotometer additionally enables the quantitative determination of formaldehyde concentration [56].

Due to the prevalence of environmental pollution by formaldehyde, it is necessary to develop easy-to-use sensors to control the quality of air and products consumed by consumers. Formaldehyde detection in milk samples is possible thanks to a matrix of colorimetric sensors made of eight quantum dots of molybdenum disulfide combined with various organic reagents deposited on polyvinylidene fluoride film. After exposure to formaldehyde, the sensors present on the matrix change their color, which enables the identification of the compound based on a colorimetric signature unique to the compound [71]. Another sensor using the colorimetric method to detect formaldehyde was created. It was based on hydroxyapatite (HAP, Ca_10_(PO_4_)_6_(OH)_2_) nanomaterial modified with silver ions, which partially replaced calcium ions. Formaldehyde in an alkaline environment reduces silver ions, which leads to the precipitation of silver nanoparticles (AgNPs-HAP), which in turn changes the color of the sensor from whitish to pale yellow to dark brown depending on the concentration of formaldehyde. In order to more accurately determine the concentration of formaldehyde without analytical instruments, the ability to assess the color intensity of the sensor was developed thanks to the ImageJ software, which analyzes a photo of the sensor taken with a smartphone [72]. The next sensor, based on o-phenylenediamine (OPD) substrate on MnO_2_ nanosheets, enabled the detection of formaldehyde in air, food samples, and beer using a fluorometric method. MnO_2_ nanoparticles catalyze the oxidation of OPD to 2,3-diaminophenazine (DAP), which, when excited with UV light at a wavelength of 560 nm, shows a bright yellow fluorescence, which formaldehyde extinguishes by binding to OPD, preventing its oxidation to DAP [54].

Studies are underway to use other methods of electrochemical detection of formaldehyde. By using the aerosol-assisted chemical vapor deposition technique, an electrochemical sensor was created consisting of fluorine-doped tin oxide (FTO) conducting glass on which nanostructured nickel thin films were placed, acting as an electrode on which formaldehyde is oxidized, which can be detected by performing CV [73]. Another option for detecting formaldehyde in the air is the use of an electrochemical sensor in the form of a field-effect transistor. Two silver electrodes were placed on SiO_2_/Si (100) substrate: source (S) and drain (D). Then, using the chemical bath deposition method, previously synthesized ZnO nanosheets (NSs) were deposited on the substrate and acted as a channel between the source and drain electrodes. A counter electrode was used to carry out the experiment. The reduction of ZnO NSs by formaldehyde leads to an increased movement of electrons, and thus an increase in the current flow [74]. An improvement in formaldehyde detection efficiency and an increase in the resistance of electrochemical sensors to interference by other substances can be achieved by adding a few oxyphilic metal atoms to Pd catalysts. The effect of the modification is the reduction of binding of carbon monoxide, a by-product of formaldehyde oxidation, on the surface of electrocatalysts. In a study, researchers designed an electrochemical sensor for formaldehyde detection using Cr-doped Pd metallene. The prepared scenarios assume the use of the sensor to monitor indoor and outdoor air and enable the assessment of food and breath monitoring in terms of formaldehyde content [75]. For the linear range and the detection limits of MnO_2_/OPD, BSA/FDH/CNT-Fe_3_O_4_/ITO, FDH/AuNPs, and Ni/FTO, see Table 3. 

### 2.3. Pentachlorophenol

#### 2.3.1. Pentachlorophenol Overview

Pure pentachlorophenol (C_6_Cl_5_OH, CAS 87-86-5, PCP) is a colorless crystal, relatively volatile, and slightly soluble in water, which does not occur naturally. In the past, PCP was widely used in algicides, bactericides, fungicides, herbicides, insecticides, and molluscicides, but since 1984, most developed countries have restricted the use of this chemical, especially in agricultural and domestic applications. Now, PCP is mainly used as a wood preservative for utility poles, railroad ties, and wharf pilings [76,77].

People can be exposed to PCP through occupational or community exposure. The former can occur during the production of PCP and formulations containing PCP, during mixing or spraying of PCP-containing formulations for agricultural use or the treatment of wood products with PCP-containing formulations, or during handling of or contact with the treated wood products [77]. Community exposure results from proximity to PCP-treated wood products, and from food, land, air, and water contaminated with PCP. Pentachlorophenol is a persistent organic pollutant, and the population may be exposed long after PCP use has been ceased [77]. 

Occupational limits for pentachlorophenol in air are set in many countries. The 8 h time weighted average (TWA) concentration limit of PCP is set at 0.5 mg/m^3^ in countries such as the USA, UK, Sweden, Spain, Poland, and Japan, 0.05 mg/m^3^ in Denmark, and 0.3 mg/m^3^ in China [78]. 

In 2019, the IARC changed the classification of pentachlorophenol from group 2B (possibly carcinogenic to humans) to group 1 (carcinogenic to humans) because of sufficient evidence in humans for causing non-Hodgkin lymphoma [77]. The relationship between PCP exposure and leukemia has not been confirmed to date. The results of studies on the relationship between exposure to pentachlorophenol and the development of leukemia are inconclusive. In a study evaluating residential exposure to polychlorinated biphenyls and organochlorine pesticides, authors observed no significant positive association between PCP exposure and ALL in children [79]. In another study, childhood leukemia and brain cancer risks were not increased among children of sawmill workers [80]. However, in one study the authors concluded that exposure to PCP before conception might increase the risk of childhood leukemia [81]. Interesting results have been reported in population studies of people living in areas contaminated with PCP [82,83]. PCP has been used to kill schistosome intermediate host snails which were epidemic mainly in China’s southern provinces across the Yangtze River watershed and areas on the south of the Yangtze River since the 1960s. The extensive use of PCP in these areas has led to significant contamination. In studies examining the population of these areas, it turned out that it is characterized by an increased risk of many types of cancer, including leukemia. For possible leukemia-inducing mechanisms of action of PCP, see Table 2

#### 2.3.2. Sensors for Pentachlorophenol Detection

There are many methods for the analytical detection of pentachlorophenol, including gas chromatography–mass spectrometry or high-performance liquid chromatography [77]. As mentioned earlier, these methods are often expensive, time-consuming, and complicated to use. For this reason, efforts are being made to develop sensors to monitor PCP concentrations.

One of the most popular strategies in the construction of sensors is the use of the phenomenon of electrochemiluminescence (ECL). ECL is a phenomenon that occurs when special molecules, known as luminophores, undergo redox reactions to form excited species that have the ability to emit light [84]. This method gained interest due to many advantages such as the ability to operate without external excitation energy, low cost, and high sensitivity and selectivity [84,85]. 

One of the possibilities is the construction of sensors based on semiconductor nanocrystals, e.g., CdS or ZnO [85,86,87]. In recent years, due to the need for ultra-sensitive tests for the detection of toxic substances, the strategy of signal amplification using biofunctional nanomaterials has attracted much attention [88]. The ECL intensity could be enhanced by certain substances. In one study it was reported that an electrochemiluminescence sensor for the PCP detection, based on the ECL amplifying behavior of graphene quantum dots–CdS nanocrystals (GQDs-CdS NCs), was constructed. The authors emphasize that using GQDs resulted in a 5-fold enhanced ECL intensity compared to the CdS NCs alone. The mechanism of operation of the device consists in the formation of excited states of CdS* and emission of light in an aqueous solution. PCP works as an inhibitor of ECL intensity. When PCP is added, it reacts with excited states of CdS* and is oxidized, which reduces the amount of CdS* and thus decreases the ECL intensity [85].

With the development of materials science, attempts have been made to improve the internal properties of GQDs. One of the proposed solutions was to modulate their properties through chemical doping and modification. Nitrogen atoms were widely used for this purpose, which resulted in the creation of nitrogen-doped GQDs (NGQDs) [89]. They have gained wide interest due to their advantages such as superior electrocatalytic activity, optical properties, and biocompatibility [90]. One paper presented the possibility of using NGQDs in the detection of pentachlorophenol, where graphene oxide (GO) was also used as immobilization support. The nanocomposites obtained in this way were tested in phosphate-buffered solutions of various pH levels, with and without K_2_S_2_O_8_ as a co-reactant, in order to evaluate ECL behaviors. Strong ECL light emission was observed in the presence of a co-reactant, suggesting that the reaction with S_2_O_8_^2−^ was responsible for the formation of excited NGQDs (NGQDs*). Next, the behavior of the sensor against increasing concentrations of PCP was investigated. The addition of PCP dramatically reduced the ECL intensity, which was explained by the oxidation of PCP on the NGQD-GO surface by radicals. The reduction in the number of radicals led to a reduced production of excited-state NGQDs*, which were responsible for light emission [89]. 

Quantum dots have been used as the luminophore of ECL sensing systems, but they have some drawbacks such as weak stability, due to their easy aggregation in aqueous solutions, or low ECL intensity. The possibility of the use of one-dimensional NWs as luminophores in ECL sensing systems was investigated. In that study, reduced graphene oxide (rGO) was used as a platform for immobilizing cadmium sulfide NWs (CdS NWs). Moreover, it appeared that not only could rGO act as the immobilization support, but it could also enhance the ECL signal intensity. Further, this signal was amplified by the addition of a co-reactant—S_2_O_8_^2−^. The sensor in question operates thanks to the creation of the excited state CdS*^+^ species which could emit light, and by adding PCP this emission is inhibited [86].

One study investigated the possibility of using zinc oxide (ZnO_2_) for the detection of PCP. The authors emphasized that ZnO_2_ nanocrystals have extraordinary detection properties; however, their disadvantages, such as weak stability or ease of aggregation, translate into difficulty when it comes to using them. In order to overcome this problem, the authors decided to introduce ZnO nanocrystals on nitrogen-doped graphene (N-GR). The ECL intensity of this sensor was enhanced by 4.3-fold compared to ZnO/GR. Next, the behavior of the sensor towards PCP was investigated. Like in the works mentioned earlier [85,86,89], in the presence of PCP there is a decrease in ECL intensity due to the depletion of excited particles—in this case ZnO* [87].

In one study, an electrochemical sensor for PCP was constructed based on the modification of a glassy carbon electrode (GCE) with silver-reduced graphene oxide nanocomposites (AgNPs-rGO). The authors emphasize that silver nanoparticles have excellent electrocatalytic properties, but the disadvantage of noble metal nanoparticles is their tendency to aggregate and oxidize. Therefore, it was decided to create a hybrid of silver nanoparticles and graphene oxide, which acted as a support material. Then, the electrochemical properties of the obtained sensor were tested by measuring the charge-transfer resistance (Rct). It turned out that the Rct value for AgNPs-rGO/GCE was lower than for the control samples, and this indicated that silver nanoparticles reduced the impedance and improved the electron-transfer rate of the material. Then, the cyclic voltammetry of PCP was measured on AgNPs-rGO/GCE. It turned out that the peak current was significantly enhanced for AgNPs-rGO/GCE, compared to control samples, confirming the sensitivity of the sensor to PCP. The behavior of the sensor towards real samples was also tested, obtaining a low detection limit [91].

In another study an electrochemical sensor based on a titania/silica hybrid xerogel modified with 4-methylpyridine (_4_-Pic), named TiSi_4_Pic^+^CL^−^, was reported. Mixed titanic/silica oxides have potential to be applied in constructing electrochemical sensors due to their large surface area, tunable pore size, and diverse structure, but the aforementioned properties required improvement. By anchoring cobalt (II) phthalocyanine (CoTsPc) in TiSi_4_PicCL^+−^, greater electroanalytical sensitivity over other sensors built with these materials alone was achieved. The sensor constructed in this way was supposed to lead to the oxidation of PCP. The reaction proposed by the authors proceeded with the release of two moles of protons and two moles of electrons [92].

Another study investigated the possibility of using metal–organic frameworks (MOFs) to create a PCP sensor, one type of MOFs being zeolitic imidazolate frameworks (ZIFs). In this work, ZIF-derived mesoporous carbon material (HZC) was used for PCP detection. It was placed on the surface of a screen-printed carbon electrode (SCPE). The sensor constructed, named HZC/SPCE, was tested with and without the presence of PCP. An increased peak current was observed in the presence of PCP. This indicated an ongoing oxidative process leading to the conversion of PCP into tetrachloroquinone which enabled the detection of PCP [93].

The phenomenon of fluorescence can also be used to detect toxic compounds. One paper presents the construction of a fluorescent sensor based on nitrogen (N)- and sulfur (S)-co-doped carbon dots (NSCDs) prepared by hydrothermal synthesis using crawfish shells. After the sensor was prepared, its fluorescent properties were tested—the fluorescence spectrum of NSCDs was recorded at an excitation wavelength of 360 nm, while the maximum fluorescence intensity was reached at 200 °C. By adding H_2_O_2_ and horseradish peroxidase (HRP), the reaction occurs, producing hydroxyl radicals. They caused the oxidation of the NSCDs, and thus the quenching of the fluorescence. The addition of PCP increased the fluorescence intensity again. This was because PCP molecules competed with NSCDs for hydroxyl radicals, which in turn led to a reduction in the oxidation of NSCDs by these highly reactive particles. The resulting NSCDs@HRP/H_2_O_2_ system for PCP detection reached a limit of detection of 2.30 µM [94]. For the linear range and the detection limits of GQDs-CdS NCs, ZnO/N-GR, NGQDs-GO, AgNPs-rGO/GCE, and CPE/TiSi-P/CoTsPc, see Table 3.

### 2.4. Styrene

#### 2.4.1. Styrene Overview

Styrene (C_6_H_5_CH=CH_2_, CAS 100-42-5) is an aromatic hydrocarbon with an alkene side chain, and a colorless and volatile derivative of toxic benzene [95,96]. The main metabolite of styrene is styrene 7,8-oxide, formed in the process of epoxidation of the alkene side chain [97,98]. Due to its ability to polymerize, styrene is widely used in the chemical industry for the production of plastics, resins, and synthetic rubbers [99]. Styrene is released into the atmosphere during the production and combustion of styrene polymers, lamination, printing, and photocopying. It is present in car exhaust fumes and cigarette smoke [97,100], and can be present in tap water and river water [101]. Styrene oligomers, by-products of the polymerization process, can migrate from polystyrene food packaging into food [102]. The WHO, in the guidelines for Europe, established that the concentration of styrene in the air should be kept below 0.26 mg/m^3^ (weekly average) and 70 μg/m^3^ (30 min average) [103].

Styrene enters the human body mainly in the process of inhalation through the respiratory tract; additionally, small amounts of styrene penetrate the body through the skin, and they can be consumed by humans together with food and drink [100]. The main metabolic pathway of absorbed styrene involves its oxidation by monooxygenases to styrene-7,8-oxide [98]. The research results showed that styrene 7,8-oxide binds to DNA to form adducts [98,99]. The IARC classified styrene and styrene-7,8-oxide as probable human carcinogens (group 2A) [104]. For possible leukemia-inducing mechanisms of action of styrene, see Table 2.

#### 2.4.2. Sensors for Styrene Detection

Styrene is a VOC that can be detected by photo-ionization detectors, electrochemical cells, and metal-oxide-based chemiresistive sensors, although the main disadvantage of these sensors is low selectivity and possible cross-reactions [105,106]. Analytical methods of styrene detection (gas chromatography with flame ionization, extractive Fourier transform infrared spectrometry) are characterized by high selectivity, but require the use of expensive and complicated instruments [104]. Therefore, research is being carried out on sensors that would be characterized by high selectivity, sensitivity, miniaturization, and a short response time.

One of the possibilities for detecting styrene in the environment is the use of a luminescent sensor made of a terbium-based metal–organic framework (Tb-MOF). The MOF is composed of Tb^3+^ metal ions linked by trimesic acid organic ligands. Upon excitation with ultraviolet light, Tb^3+^ ions exhibit green fluorescence. VOCs had various effects on the intensity of fluorescence emitted by the sensor, with styrene significantly reducing it, gradually leading to complete extinction of green fluorescence, which made it possible to distinguish it from other VOCs [106]. Additional advantages of gas sensors based on MOFs are their large surface area and porosity, which facilitate gas absorption [107]. Another sensor for detecting styrene is based on a lanthanide-modified supramolecular organic framework. Using the EPD method, a luminescent Eu@SOF film was prepared. Europium ions (Eu^3+^) exhibit a red fluorescence that is gradually extinguished upon exposure to styrene. The sensor detects gaseous styrene and styrene present in water [101]. For the linear range and the detection limits of Eu@SOF film, see Table 3.

### 2.5. Diazinon

#### 2.5.1. Diazinon Overview

Diazinon (*O*,*O*-diethyl *O*-2-isopropyl-6-methylpyrimidin-4-yl phosphorothioate, C_12_H_21_N_2_O_3_PS, CAS 333-41-5) is the common name of an organophosphorus pesticide that does not occur naturally in the environment. Its pure form is colorless and odorless oil, but formulations containing 85–90% diazinon appear as pale to dark brown liquid. It is slightly soluble in water and completely miscible with common organic solvents [108]. This organophosphate insecticide has been widely used in agriculture to control crop pests by attacking their nervous system [109]. The main mechanism of action of diazinon is the inhibition of acetylcholinesterase, which leads to the accumulation of acetylcholine in synapses and, as a result, uncontrolled nerve impulses and the death of the organism [110].

Excessive use of this pesticide can result in its accumulation in the environment, causing pollution. In this case, diazinon residues may accumulate in water and agricultural crops, thus posing a risk to human health. This is why limits on the concentration of diazinon in food products have been introduced, e.g., in the EU, the maximum residue level for diazinon has been set in the range of 0.01–0.05 mg/kg for fruit and vegetables [111].

The main ways of entry of diazinon into the human body are dermal exposure resulting from occupational practices and oral exposure from the diet. After absorption, diazinon is metabolized in the liver by several enzymes: cytochrome P450 (CYP), paraoxonases, and carboxylesterases. Its metabolites can have a toxic effect on the human body by inhibiting several serine hydrolase enzymes, including butyrylcholinesterase, acetylcholinesterase, and carboxylesterases [112]. The toxicity of diazinon has been investigated in numerous animal studies. In laboratory animals exposed to diazinon, the following adverse events have been shown: cholinergic effects, liver hypertrophy, gastrointestinal tract issues (congestion, erosion, and hemorrhage), decreased testicular weight, sperm count, and quality, decreased lung weight, and cardiotoxicity [112]. This pesticide has also attracted the attention of many researchers due to the suspicion of its carcinogenicity. Following the results of multiple carcinogenicity studies in animals and humans, the IARC has decided to place diazinon in group 2A (probably carcinogenic to humans) due to limited evidence for carcinogenicity in animals and humans [112]. For possible leukemia-inducing mechanisms of action of diazinon, see Table 2.

#### 2.5.2. Sensors for Diazinon Detection

Analytical methods for detecting diazinon include high-performance liquid chromatography, liquid chromatography coupled with mass spectroscopy, gas chromatography–mass spectrometry, capillary gas chromatography, electrochemical analysis, and colorimetric methods [113]. Due to the limitations of the aforementioned methods, work is being undertaken to develop sensors for detecting diazinon. They include, for instance, the use of acetylcholinesterase or MIP technology, which seems to be a promising direction due to high efficiency and the ability to analyze samples in all states of aggregation [114].

Enzymes are often used to construct sensors because of their catalytic activity towards specific compounds. A biosensor for the detection of diazinon based on acetylcholinesterase (AChE) was developed. The enzyme was placed in a pH-sensitive rGO layer, referred to as a WE. In order to improve the sensitivity of the biosensor, 4-aminobenzoic acid was added to the rGO layer. The WE was placed on a polyethylene terephthalate board. Diazinon blocks the catalytic activity of AChE, impairing its ability to decompose acetylcholine into choline and acetic acid, which is perceived as the change in the potential of the WE [115]. One of the disadvantages of using enzymes to construct sensors is the need to provide them with appropriate physicochemical conditions of the reaction environment. Work is underway to produce sensors with similar specificity without the use of enzymes. An enzyme-free ECL sensor for detecting diazinon was developed. A suspension of GO was dropped on the surface of the glassy carbon electrode. Then AgNPs were placed on the electrode. The next step was to place ruthenium nanoparticles (RuNPs), acting as a luminophore, on a nanocomposite-modified electrode (AgNPs/GO/GCE). As a co-reactant, boron nitride quantum dots (BNQDs) were used, which increased the signal emitted by the luminophore. Diazinon, interacting with BNQDs, extinguishes the luminescence emitted by the sensor [116].

One of the ways to maintain the appropriate specificity of the sensor without the use of enzymes is MIP. A group of researchers developed an electrochemical sensor made of a carbon paste electrode (CPE) modified with nanoparticles of diazinon-imprinted polymer (nano-MIP-CP). The use of MIP made it possible to create a sensor with high selectivity by giving it the ability to recognize diazinon on the basis of an “artificial receptor”. Square wave voltammetry was used as the measurement method and a reduction signal for diazinon was observed. The studies also showed that the nano-MIP-CP electrode, compared to the MIP-CP electrode, has a higher binding capacity and greater affinity for diazinon molecules due to the increased surface area [117]. Research is being conducted on optimizing the composition of the CPE to improve the properties of the sensors. Another sensor was developed with CPE-modified multi-wall carbon nanotubes (MWCNTs) and MIP. It has been shown that the sensor synthesized in this way is characterized by increased peak currents due to the increased conductivity of the sensor and the rate of transduction of the chemical signal to electrical signal by MWCNTs [118]. For the linear range and the detection limits of RuNPs/AgNPs/GO/GCE and CPE/MWCNT film, see Table 3.

### 2.6. Future Perspectives

To the best of our current knowledge, none of the reported electrochemical or optical sensors are commercially available yet. Some of them are still in the testing phase in order to optimize their functioning and reduce their production costs. The technology of creating sensors is still developing. Future directions for developing and improving these devices should include the use of new technologies to further enhance sensitivity and selectivity. A promising prospect for the future is the production of sensors based on MIP technology, due to the very high selectivity of such devices. Currently, work is being carried out on the use of MIP technology in commercial production of sensors, but there is a problem in the creation of MIP in large batches that are homogeneous in terms of affinity for the target [114]. Another important aspect is the miniaturization of sensors in the form of handy devices, as well as the possibility of connecting the software with mobile devices, which will enable the use of sensors by people unqualified to operate difficult and complex analytical devices. Another important issue is the use of environmentally friendly, biodegradable materials that are also cheap to produce, which would facilitate access to such sensors, and would also enable their large-scale production [11].

**Table 2:** Possible mechanisms of action of leukemia-inducing chemical carcinogens.

Chemical Carcinogen	Possible Mechanism of Carcinogenesis Induction	**Reference**
Benzene	Topoisomerase-IIα inhibition	[18]
	Causes oxidative stress by increasing the level of ROS	[18]
	Causes oxidative DNA damage	[19]
	Increases the risk of chromosomal aberration (translocation, aneuploidy)	[18]
	Increases the risk of forming micronuclei	[19]
	May form covalent bonds with nucleophilic sites	[18]
	Binds the metabolite of benzene to DNA and protein to form adducts	[19]
Formaldehyde	May form covalent bonds with nucleophilic sites	[41,60]
	Causes oxidative stress by increasing the level of ROS	[41,60]
	Reduces the expression of the *PRDX2* gene	[41,60]
	Reduces the expression of *GM-CSFRα* and *EPOR* genes	[61]
	Increases the risk of chromosome 7 monosomy and chromosome 8 trisomy	[62]
Pentachlorophenol	Causes oxidative stress by increasing the level of ROS	[77]
	Causes DNA damage	[77]
	Increases the risk of chromosomal aberration	[77]
Styrene	Binds the metabolite of styrene to DNA to form adducts	[98,99]
Diazinon	Causes DNA damage	[112]
	Increases the frequency of chromosomal aberrations	[112]
	Increases the risk of forming micronuclei	[112]

DNA—deoxyribonucleic acid, ROS—reactive oxygen species, PRDX2—peroxiredoxin 2, GM-CSFRα—α subunits of granulocyte–macrophage colony-stimulating factor receptor, EPOR—erythropoietin receptor.

**Table 3:** Comparison of the performance of the selected sensors with different electrode materials in detecting leukemia-inducing chemical carcinogens.

Chemical Carcinogens and Their Norms from https://www.osha.gov/ (accessed on 30 November 2020)	Sensor	Linear Range	Limit of Detection	Reference
Benzene PEL-TWA: 1 ppm REL-TWA: 0.1 ppm	SnO_2_/Rh-TiO_2_	-	77.6 ppb	[25]
	Pd-decorated TiO_2_/MoS_2_	1–100 ppm	100 ppb	[28]
	ZnO-CdO	1–200 ppm	1 ppm	[23]
	(C_4_H_9_NH_3_)_2_PbI_2_Br_2_	-	1 ppt	[31]
	IL/pNIPAAM	4–20 ppm	47 ppb	[32]
Formaldehyde PEL-TWA: 0.75 ppm REL-TWA: 0.016 ppm	MnO_2_/OPD	0.8–100 μM	6.2 × 10^−8^ M	[54]
	BSA/FDH/CNT-Fe_3_O_4_/ITO	0.05–0.5 mg/L	0.05 mg/L	[55]
	FDH/AuNPs	0.25–2.0 mM	0.05 mM	[69]
	Ni/FTO	0–6.5 mM	8.3 × 10^−6^ M	[73]
Pentachlorophenol PEL-TWA: 0.5 mg/m³ REL-TWA: 0.5 mg/m³	GQDs-CdS NCs	0.01–500 ng/mL	3 pg/mL	[85]
	ZnO/N-GR	0.5 pM–61.1 nM	0.16 pM	[87]
	NGQDs-GO	0.1–10 pg/ml	0.03 pg/mL	[89]
	AgNPs-rGO/GCE	0.008–10.0 μM	0.001 μM	[91]
	CPE/TiSi-P/CoTsPc	0.99–4.21 μM/L	0.029 μM/L	[92]
Styrene PEL-TWA: 100 ppm REL-TWA: 50 ppm	Eu@SOF	10^−7^–10^−2^ M	0.20 ppm	[101]
Diazinon PEL-TWA: - REL-TWA: 0.1 mg/m^3^	RuNPs/AgNPs/GO/GCE	3.0 × 10^−15^–6.5 × 10^−9^ M	9.5 × 10^−16^ M	[116]
	CPE/MWCNTs	5 × 10^−10^–1 × 10^−6^ M	1.3 × 10^−10^ M	[118]

PEL-TWA—8 h total weight average permissible exposure limit, REL-TWA—8 h total weight average recommended exposure limit, SnO_2_/Rh-TiO—tin (IV) oxide with rodium and titanium (IV) oxide catalytic overlayer, Pd-decorated TiO_2_/MoS_2_—titanium (IV) oxide/molybdenum disulfide decorated with palladium, ZnO-CdO—zinc (II) oxide with cadmium (II) oxide, (C_4_H_9_NH_3_)_2_PbI_2_Br_2_—organic–inorganic layered perovskite compound, IL/pNIPAAM—ionic liquid encapsulated into a poly(*N*-isopropylacrylamide) matrix, MnO_2_/OPD—manganese (IV) oxide nanoparticles on o-phenylenediamine, BSA/FDH/CNT-Fe_3_O_4_/ITO—bovine serum albumin solution/formaldehyde dehydrogenase/carbon nanotubes and iron(II,III) oxide nanocomposites/indium tin oxide, FDH/AuNPs—formaldehyde dehydrogenase/gold nanoparticles, Ni/FTO—nickel films placed on fluorine-doped tin oxide, GQDs-CdS NCs—graphene quantum dots with cadmium sulfide nanocrystals, ZnO/N-GR—zinc oxide nanocrystals placed on nitrogen-doped graphene, NGQDs-GO—nitrogen-doped graphene quantum dots with graphene oxide, AgNPs-rGO/GCE—silver-reduced graphene oxide nanocomposites/glassy carbon electrode, CPE/TiSi-P/CoTsPc—carbon paste electrode/titania/silica hybrid xerogel modified with 4-methylpyridine/cobalt (II) phthalocyanine, Eu@SOF—europium supramolecular organic framework, RuNPs/AgNPs/GO/GCE—ruthenium nanoparticles/silver nanoparticles/graphene oxide/glassy carbon electrode, CPE/MWCNTs—carbon paste electrode/multi-wall carbon nanotubes.

## 3. Conclusions

The article presents some of the recently designed electrochemical sensors and biosensors that facilitate the detection of compounds (i.e., benzene, formaldehyde, pentachlorophenol, diazinon, and styrene) which may contribute to the development of leukemia as a result of exposure to them in the environment or diet. In addition, the paper has covered such themes as the methods of operation of all sensors as well as the linear range and the detection limits for specific ones. The use of sensors in monitoring the presence of toxic compounds in the environment has numerous advantages. High selectivity, the speed of analysis, and repeatability enable one to control the concentration of toxic substances in real time. The development of miniaturized and easy-to-use sensors makes it possible for unqualified people to use them in everyday life. Moreover, an additional advantage is the use of biodegradable and non-toxic materials in their production.

## Figures and Tables

**Table 1:** Leukemia-inducing chemical carcinogens listed by IARC.

Type of Leukemia	Chemical Carcinogens in the Environment with Sufficient Evidence in Humans	Chemical Agents in the Environment with Limited Evidence in Humans
AML	Benzene Formaldehyde Tobacco smoking	-
Childhood AML	-	Benzene Tobacco smoking (parental)
ALL	-	-
Childhood ALL	-	Tobacco smoking (parental)
CML	Formaldehyde Tobacco smoking	Benzene
CLL	-	Benzene Ethylene oxide
Leukemia: all combined	1,3-Butadiene Rubber manufacturing industry	Diazinon Nitrogen mustard Petroleum refining Styrene

AML—acute myeloid leukemia, ALL—acute lymphoblastic leukemia, CML—chronic myeloid leukemia, CLL—chronic lymphocytic leukemia.

## Data Availability

Not applicable.
